# Colorimetric Nanoplasmonics to Spot Hyperglycemia From Saliva

**DOI:** 10.3389/fbioe.2020.601216

**Published:** 2020-12-07

**Authors:** Paolo Donati, Tania Pomili, Luca Boselli, Pier P. Pompa

**Affiliations:** Nanobiointeractions and Nanodiagnostics, Istituto Italiano di Tecnologia, Genova, Italy

**Keywords:** plasmonics, colorimetric sensors, hyperglycemia, gold nanoparticles, reshaping

## Abstract

Early diagnostics and point-of-care (POC) devices can save people’s lives or drastically improve their quality. In particular, millions of diabetic patients worldwide benefit from POC devices for frequent self-monitoring of blood glucose. Yet, this still involves invasive sampling processes, which are quite discomforting for frequent measurements, or implantable devices dedicated to selected chronic patients, thus precluding large-scale monitoring of the globally increasing diabetic disorders. Here, we report a non-invasive colorimetric sensing platform to identify hyperglycemia from saliva. We designed plasmonic multibranched gold nanostructures, able to rapidly change their shape and color (naked-eye detection) in the presence of hyperglycemic conditions. This “reshaping approach” provides a fast visual response and high sensitivity, overcoming common detection issues related to signal (color intensity) losses and bio-matrix interferences. Notably, optimal performances of the assay were achieved in real biological samples, where the biomolecular environment was found to play a key role. Finally, we developed a dipstick prototype as a rapid home-testing kit.

## Introduction

Point of care (POC) devices for self-monitoring of blood glucose are a life-changing technology and nowadays a norm for diabetes control. However, commercially available devices generally employ a relatively expensive hardware (reading system) and require a quite invasive sampling process (finger-pricks) involving a certain physical discomfort, especially for patients necessitating frequent measurements. It is worth mentioning that repeated finger-prick sampling also presents some risk, due to possible exposure to blood-borne pathogens and infections (Thompson and Perz, 2009). For these reasons, small implantable sensors have recently entered the clinical use for chronic patients, and different non-invasive approaches, such as near-infrared transcutaneous spectroscopy (Yadav et al., 2015), breath acetone measurements (Saasa et al., 2018), and other optical and electrical/electrochemical sensing techniques, are currently under investigation in the field (Bharathi and Nogami, 2001; Luo et al., 2004; Wu et al., 2007; Jv et al., 2010; Jiang et al., 2010; Xiong et al., 2015; Xu et al., 2017; Cao et al., 2019; Shokrekhodaei and Quinones, 2020).

An interesting alternative currently attracting tremendous interest in the diagnostic community is the use of saliva as biological source (Kaufman and Lamster, 2000; Zhang et al., 2016; Kim et al., 2019; To et al., 2020). Saliva holds a huge variety of well-known disease-related biomarkers, including glucose, representing an ideal medium for the development of non-invasive tools for self-monitoring of hyperglycemia (Elkington et al., 2014; Zhang W. et al., 2015; Zhang et al., 2016). Several studies showed good correlation between the glucose amount present in saliva and in blood (Gupta et al., 2017). However, salivary glucose was found to be ca. 100 times less concentrated (Zhang W. et al., 2015). This requires the new salivary POCs to gain a breakthrough in terms of sensitivity, while maintaining rapidity, accuracy, and clear readout. A few examples in this context have been recently reported (Elkington et al., 2014), including pioneering works on wearable devices (de Castro et al., 2019; Arakawa et al., 2020), yet many opportunities in this field remain unexplored.

In this framework, for example, gold nanoparticles (GNPs) present enormous potential for the development of a new generation of highly sensitive sensors (Liu and Lu, 2003; Luo et al., 2004; Pingarrón et al., 2008; Sperling et al., 2008; Zhang et al., 2012; Valentini and Pompa, 2013, 2016; Howes et al., 2014; Chen et al., 2016; Valentini et al., 2017; Qin et al., 2018; Quesada-González and Merkoçi, 2018; Donati et al., 2019; Hao et al., 2019; Loynachan et al., 2019; Quesada-González et al., 2019), due to their unique size-, shape-, and dispersion-state-dependent plasmonic properties, which can be exploited for the realization of “naked-eye detection” assays (Baron et al., 2005; Wang et al., 2006; Song et al., 2011; Liu et al., 2012; Zhao et al., 2016; Donati et al., 2019; Lu et al., 2019; Xu et al., 2019). Common colorimetric approaches involve target-mediated GNP aggregation/assembly strategies or growth/etching processes (Valentini et al., 2013; Rao et al., 2019). The “aggregation approach” can be very fast and sensitive, but it often requires precise NP functionalization to ensure selectivity. In addition, in biological media, the “biomolecular corona” can potentially mask the prepared functionality, often promoting non-specific interactions (i.e., NP uncontrolled aggregation) (Monopoli et al., 2012; Del Pino et al., 2014; Castagnola et al., 2017). Generally, the “growth approach” also presents some drawbacks in complex media, since the surrounding proteins and metabolites could interact with the reagents involved, while protein adsorption onto the GNPs can interfere in the nanostructure growth (i.e., inhibiting the process or even acting as shape directing agents) (Chakraborty et al., 2018; Chakraborty and Parak, 2019). Nevertheless, some very interesting examples are reported in the literature (Rodríguez-Lorenzo et al., 2012). A more promising strategy is the “etching approach” involving, in the case of glucose sensors, the glucose oxidase (GOx) enzyme. GOx is able to react specifically with glucose in complex media producing H_2_O_2_ that, via Fenton or Fenton-like reactions, transforms in the free radical form able to rapidly oxidize (and partially dissolve) the GNPs (Rao et al., 2019). A colorimetric assay for blood glucose determination using this mechanism was explored on gold nanorods and the color change was obtained following the particle corrosion and shortening in presence of glucose (Liu et al., 2013).

Here, to gain the high sensitivity necessary for rapid glucose detection at salivary concentration, we developed a novel strategy combining multibranched (spiky) GNPs, GOx, and bromine (Br^–^) mediated particle reshaping, which allows for a rapid color change without any optical density (OD) loss, typically related to NP dissolution. Furthermore, we transferred our sensing technology onto a substrate, obtaining a rapid and highly sensitive colorimetric home-testing prototype for self-monitoring of salivary glucose levels.

## Results

**FIGURE 1:**
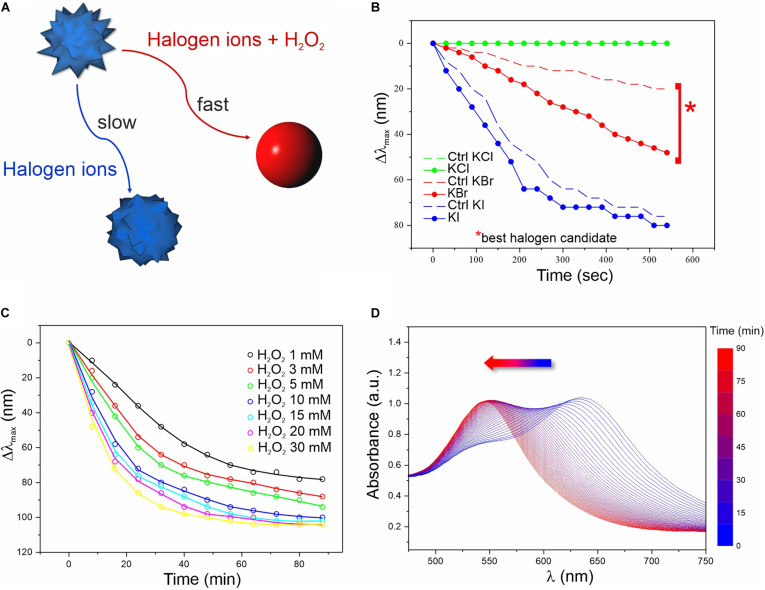
Screening of the chemical conditions for the colorimetric assay. **(A)** Schematics of the reactions involved in the MGNP morphological changes in water. **(B)** LSPR λ_max_ evolution over time related to MGNP reaction with H_2_O_2_ (3 mM) in presence of optimized concentrations of different halogens (more info in Supplementary Material). Control (Ctrl) performed in absence of H_2_O_2_. **(C)** LSPR λ_max_ evolution of MGNPs exposed to Br**^–^** (5 mM) with increasing H_2_O_2_ concentration (1–30 mM). **(D)** Absorption spectra related to the MGNP reshaping process for optimized reaction conditions (see Supplementary Material). All reactions were carried out at pH = 5.

For simplicity, basic chemical aspects and optimizations of the sensing platform were preliminarily explored in water solvent and in absence of enzyme (Figure 1). A synthetic procedure for highly responsive multibranched GNPs (MGNPs) was optimized for the current strategy, slightly modifying a previously reported method (Maiorano et al., 2011). The prepared colloidal suspensions were fully characterized (see Supplementary Material and Supplementary Figures S1, S2). The MGNPs were monodisperse, presenting an average size of about 60 nm. The selected stabilizing agent (Hepes) guaranteed high NP reactivity and colloidal stability. Concerning the particle shape, the tips represented the more sensitive units (where the lowest energy plasmon mode is mainly localized) (Rodríguez-Lorenzo et al., 2009), therefore the nanostructures were tuned to obtain multiple short tips (average length of c.a. 8 nm) to gain clear color distinction with minimal morphological changes (i.e., through preferential tips gold oxidation). Since the spiky structure is responsible of the MGNPs blue color, the NPs gradually change to the characteristic red color when becoming spherical (Kumar et al., 2007; Rodríguez-Lorenzo et al., 2011; Potenza et al., 2017).

We first evaluated the MGNP responses to different pH values (Supplementary Figure S3) and halogens (Figure 1B) in presence or absence (negative control) of H_2_O_2_. At certain conditions, H_2_O_2_ can itself oxidize GNP surface atoms, leading to etching and morphological changes. However, this process has low efficiency and normally occurs on a much larger timescale (several hours) compared to our aimed few-minute response (Tsung et al., 2006). Therefore, we evaluated the effect of three different halogens (I^–^, Br^–^, and Cl^–^) potentially able, in acidic conditions, to boost the oxidation process and promote a quick color change (Yuan et al., 2015; Zhang Z. et al., 2015; Zhu et al., 2015; Cheng et al., 2019).

As expected, in presence of H_2_O_2_ and Br^–^ (2:1 molar ratio), MGNPs presented faster LSPR blue-shift when lowering the pH (see Supplementary Figure S3). Nevertheless, all tested conditions showed a remarkable *λ_*max*_* shift within 10 min, and pH = 5 was selected as the optimal condition for the enzyme activity as well as NP stability (Supplementary Figure S3B). Concerning the halogen ions tested, bromine resulted the best candidate, promoting a fast (within 10 min) and wide LSPR shift in presence of a relatively low H_2_O_2_ concentration (together with a relatively small shift for the control), while iodine ions led to drastic blue-shift, independently of H_2_O_2_ (Figure 1B). No interesting effects were observed using chlorine ions for this platform. In presence of Br^–^ at established concentration, increasing the amount of H_2_O_2_ led to faster morphological changes (Figure 1C and Supplementary Figure S4), but at high concentrations (≥10 mM) harsher etching involved also some OD loss (see Supplementary Figure S5). However, within the concentration range of interest (1–3 mM), irreversible corrosion of the nanostructure was avoided (or very limited) even on the long-term (see the plateau in Figures 1C,D), while keeping relatively fast spectral changes. When carefully dosing the MGNPs/Br^–^/H_2_O_2_ stoichiometry, we could achieve a final absorbance as intense as the starting one (Figure 1D), and an almost linear LSPR evolution pathway with minor intensity differences. However, in water, the presented platform has significant limitations, due to some uncontrolled etching occurring in the control, with relatively small variations in the reagents molar ratio.

**FIGURE 2:**
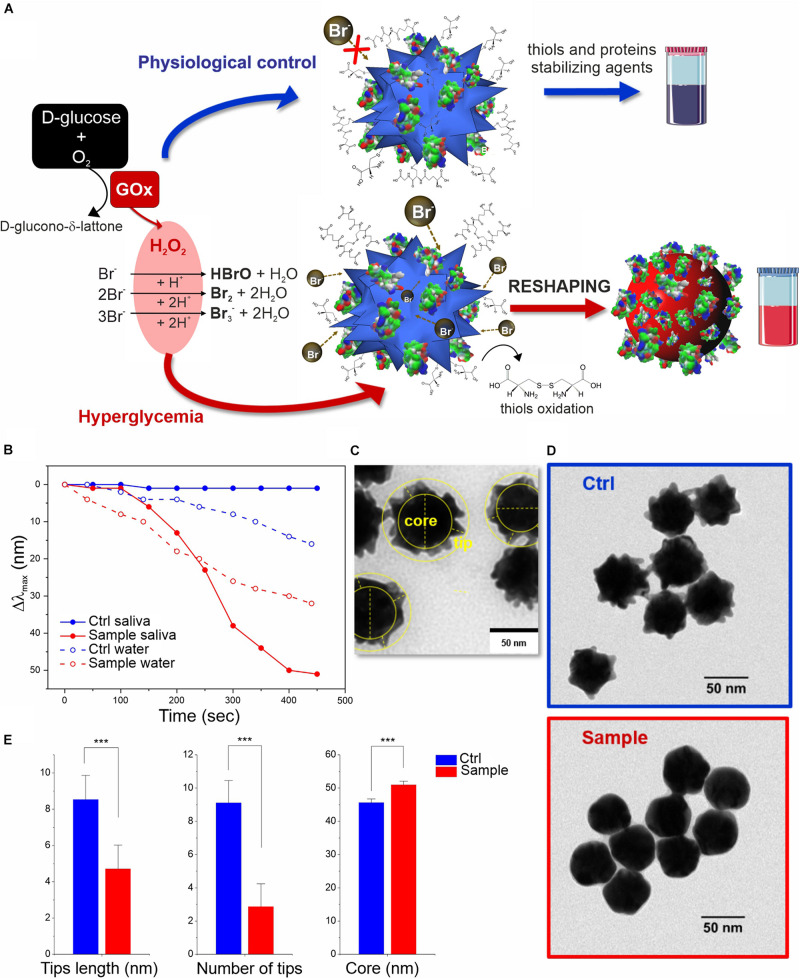
*In vitro* colorimetric assay using MGNPs suspended in saliva: **(A)** schematics of the mechanisms involved; **(B)** LSPR Δλ_max_ evolution over time related to the reshaping in saliva and in water (the two model platforms performance are compared at their own Br^–^ optimized conditions while involving the same particles number, and H_2_O_2_ final concentration, 2 mM). TEM micrographs related to the MGNPs before **(C)** and after **(D)** the assay with non-supplemented saliva (blue) and with glucose supplement to simulate hyperglycemia condition (red). **(E)** Statistical TEM analysis measuring tips (numbers and lengths) and “core” dimensional variation between control and sample nanostructures (*p* < 0.001 in all cases). Statistical significance was determined by Mann-Whitney test (***p < 0.001). See experimental details in Supplementary Material.

Adapting this platform to real working conditions in saliva, employing glucose spikes, GOx enzyme, and a large excess of Br^–^ (see scheme in Figure 2A) resulted in a strikingly better performance than the previous one. The system was so stable that we could employ much larger Br^–^ concentration than in water, without affecting the control. Proteins and metabolites present in saliva can rapidly coat the MGNPs forming the so-called biomolecular corona, which can act as an organic shield protecting and stabilizing the surface atoms (see Supplementary Figure S6). In particular, proteins (i.e., mucin) promote higher colloidal stability limiting the surface availability by steric hindrance, while salivary thiols (i.e., cysteine, glutathione and others) can act as surface ligands (Yang et al., 2007; Boselli et al., 2017). These factors together firmly preserve the MGNP shape even in presence of a large excess of Br^–^ that otherwise would lead to uncontrolled fast etching and immediate color change even in absence of glucose. Indeed, the saliva medium allowed for a significant extension of the dynamic range of the assay along with a faster response, meaning that resolution and sensitivity were also strongly improved (see Figure 2B). Analyzing the physiological control (no added glucose), no significant spectral changes were observed, indicating a better resolution of the nanosensor in saliva compared to non-biological media (see Figures 1B, 2A,B). This is a key point since it excludes the possibility of false positives, due to spontaneous color changes of the MGNPs in the test timeframe (10–20 min); furthermore, it guarantees a faster naked-eye recognizable color distinction, due exclusively to the glucose present in saliva. The presence of non-physiological glucose concentrations in saliva (≥2 mM), reproducing hyperglycemia conditions, indeed promoted a fast spectral change with a large blue-shift of the LSPR *λ_*max*_*, and no significant OD loss (Supplementary Figure S7).

The molecular mechanism underlying the sensing strategy can be divided in two phases, one involving the oxidant species production and a second in which such species trigger a series of reactions, leading to MGNP morphological changes. The first part is quite well-known, at least in its separate steps: the glucose/GOx-generated H_2_O_2_ reacts with the Br^–^ present in stoichiometric excess (even if part of it might be sequestrated by saliva components), producing Br_2,_ Br_3_^–^ and HBrO (the latter eventually converting to Br_2_) (Yuan et al., 2015; Zhu et al., 2015).

In the second phase, the bromine oxidant species can interact with MGNPs through multiple pathways: beyond direct gold surface oxidation, the reaction mechanism responsible for the reshaping process is likely to involve the biomolecular corona (including salivary proteins and thiols). It may be envisaged that the surface atoms of the MGNP tips are less protected by the protein layer, thus being more prone to oxidation than the particle core. The structural changes are also promoted by oxidation of the salivary thiol ligands by Br_2_ (Wu et al., 1996), leading to an unanchored surface (with high tension) that can immediately start rearranging. This reshaping process is probably additionally boosted by the Br^–^ excess coordination (Jang et al., 2012). Furthermore, a series of gold oxidation/reduction reactions (promoted by Br_2_ and R-SH species, respectively) can also promote gold atoms migration from surface convexity to the concavity (from higher to lower surface potential), leading to the more thermodynamically stable spherical shape (Xia et al., 2009). In this case, R-SH species would play a double role, protecting the shape in absence of glucose but also supporting the reshaping (instead of the etching) in presence of hyperglycemic conditions.

The reshaping process in saliva was also analyzed by TEM (Figures 2C–E and Supplementary Figure S8), confirming no significant differences in the morphology of MGNP control (in absence of glucose supplement) with respect to primary particles, while an evident smoothening of the nanostructure surface was observed in hyperglycemic conditions, obtaining quasi-spherical GNP shape. Furthermore, statistical geometrical measurements (Figure 2E) showed a final size distribution compatible with a redistribution of the MGNP gold atoms onto the surface concavities, with a negligible loss of the metal atoms. The “core size” increase of the nanostructure, indeed, can be considered as another evidence in favor of gold atoms rearrangement process and particle reshaping, against irreversible “MGNPs dissolution” (see Figures 2C,E). The described results demonstrated that we were able to achieve sensitive glucose detection without losing the optical signal (color intensity), which is a significant technological advancement, especially aiming at “naked-eye” detection.

In order to realize a portable home-testing device prototype, the sensing platform was transferred onto a solid substrate. Among the different materials tested (including cellulose acetate, nitrocellulose, PVDF), a porous nylon membrane was selected, presenting well structured (ordered) surface and a good balance between wettability and hydrophobicity (see Supplementary Figure S9), enabling stable adsorption of MGNPs. By placing the membrane in a syringe filter holder, we could homogeneously immobilize a reproducible particle amount in a few seconds by simple injection. Additionally, few microlitres of the enzyme were also deposited to have all the reagents, otherwise suffering for long-term stability, dryed on the substrate, and ready for the analysis (see Supplementary Material).

The use of a solid substrate, led to a significant improvement of the reagents stability. While colloidal dispersions of MGNPs in water lose their morphological and plasmonic properties overtime, the on-substrate assay showed excellent stability up to 6 months, meaning that also the enzyme functionality was maintained (see Supplementary Figure S10). An additional advantage is that, even after glucose testing, the “test strip” could be stored, keeping the outcome unaltered for ≥6 months.

**FIGURE 3:**
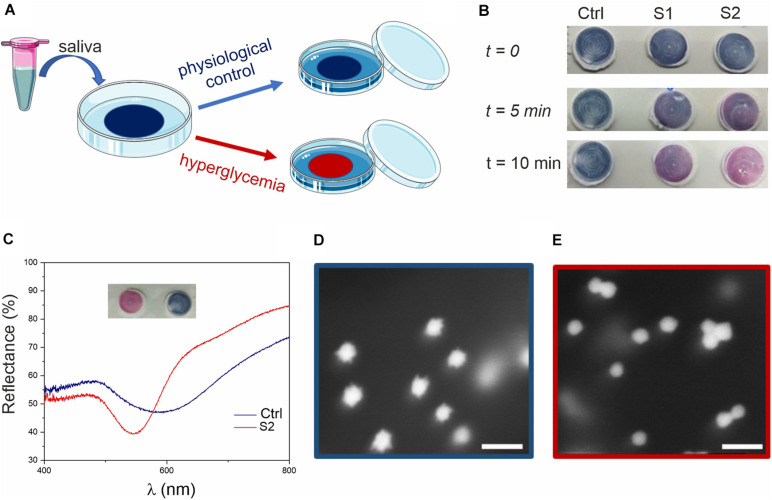
Glucose colorimetric assay prototype for home-testing of saliva samples. **(A)** Schematics of the assay. The blue color of the membrane (due to the MGNPs on its surface) changes to pink/red if the saliva sample presents hyperglycemic conditions. **(B)** Colorimetric assay applied to human saliva (ctrl) and to human saliva supplemented with glucose at the concentration of 15 mg/dL (0.8 mM, S1) and 30 mg/dL (1.6 mM, S2). **(C)** Reflectance spectra of the membrane substrates, related to ctrl and S2 (picture in inset), showing a clear blue-shift (from λ = 600–550 nm). HR-SEM micrographs of **(D)** ctrl assay (blue) and **(E)** sample S2 (red) assay (scale bars: 100 nm).

Saliva samples were tested with this dipstick-like colorimetric assay prototype as depicted in the schematics reported in Figure 3A. A small amount (20 μL) of glucose supplemented saliva (with glucose above physiological concentrations) was sufficient to trigger a rapid color change from blue to red (Figure 3B) within 5–15 min, depending on the glucose concentration. Further controls related to the assay are reported in Supplementary Figure S11.

After assessing hyperglycemic saliva and non-supplemented saliva (control), the substrates were analyzed by using reflectance spectroscopy (Figure 3C) and scanning electron microscopy (SEM, Figures 3D,E). From the reflectance spectra, glucose supplemented saliva appeared to be ca. 50 nm blue-shifted compared to the control (consistent with the data observed in suspension), with the curves minima corresponding to the particles LSPR. From SEM imaging, we could observe the nanostructure morphology directly on the membrane, confirming the reshaping process also on the substrate, with spherical GNPs on the test membrane after exposure to hyperglycemic saliva.

The assay prototype was finally optimized for analysis on real samples, considering that physiological glucose concentration in saliva is commonly <2 mg/dL (<130 mg/dL in human serum) while it is ≥4 mg/dL for hyperglycemic condition (≥160–200 mg/dL in human serum) (Abikshyeet et al., 2012). It is important to stress that the correlation between hematic and salivary glucose is not based on a constant ratio over the whole range of concentrations (Abikshyeet et al., 2012).

For this reason, aiming at an ON/OFF response as an alarm bell for healthcare, we set our threshold about ≥4 ± 0.5 mg/dL. For salivary glucose concentrations above this range values, the assay must produce an evident color change.

To numerically estimate the colorimetric changes of our device and perform statistical data analysis, RGB coordinates of the substrate were acquired using a smartphone app (see Supplementary Material).

**FIGURE 4:**
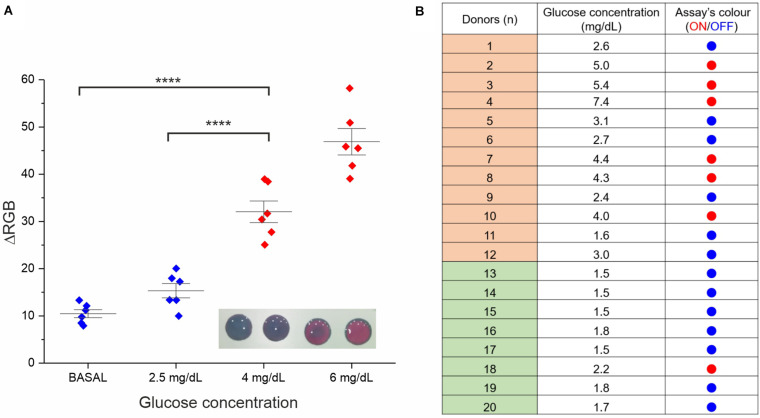
Assay performance analysis in real saliva samples. **(A)** Validation of the RGB-based readout. Six saliva samples were involved in this study (the average of two experimental replicates is reported). Besides the basal glucose concentration, all samples were normalized through glucose spike at 2.5, 4, and 6 mg/dL, the RGB coordinates values were measured at *t* = 0 and 15 min with a smartphone app (ColorGrab), and the ΔRGB was calculated. Statistical significance was determined using a one-way ANOVA and Tukey’s multiple comparison test (****P < 0.0001). A representative image of the assay at the four different concentrations is reported in the inset. **(B)** Assay results for clinical saliva samples from both diabetic subjects (donors 1–12) and healthy subjects (donor 13–20). The “ON” outcome (red dots) refers to ΔRGB ≥ 30 ± 10.

A proof-of-concept experiment was performed measuring multiple saliva samples from different healthy donors with the addition of increasing concentrations of glucose from basal physiological values (<2 mg/dL) to hyperglycemic ones (4 and 6 mg/dL). A significant difference in the color variation was observed between physiological and hyperglycemic conditions by both naked-eye and RGB analysis, identifying our colorimetric threshold as ΔRGB ≥ 30 ± 10 (see Figure 4).

ΔRGB values were also employed to measure the limit of detection (LOD). In this context, the assay performances were very good and reproducible using different independently produced devices to analyze the same saliva sample from a donor, reaching a LOD of 0.4 mg/dL (see Supplementary Figure S12), in line with the best performing glucose colorimetric sensors reported (Gabriel et al., 2016; de Castro et al., 2019). However, some variability on the LOD values was expected when analyzing saliva samples from multiple different donors (presenting some difference in the composition), resulting in a more representative average value of 1.4 mg/dL (see Supplementary Material). Such LOD value is still appropriate for our ON/OFF detection, allowing to cope with the intrinsic biological variability or real clinical samples.

Further validation of the assay prototype was performed through a small clinical study involving twenty different saliva samples, 12 from diabetic subjects and 8 from healthy subjects (see Figure 4B). The analysis was carried out using in parallel a commercial high-sensitive glucose kit (as standard reference technique) and our device. We observed good reliability of our rapid test (95%) from this screening, attesting concrete potential for future applications.

## Discussion

The nanoscale architecture of MGNPs and their sensitive plasmonic features were exploited for the development of a novel colorimetric assay for hyperglycemia, demonstrated to be effective in real saliva samples. Interestingly, the sensing platform, when operating in salivary medium, showed improved solidity and better dynamic range than in water, suggesting a proactive role of the biomolecular corona both in stabilizing the nanosensor and in promoting the reshaping process (instead of etching). The technological transfer from solution to the solid substrate finally led to the realization of a dipstick-like prototype for non-invasive self-monitoring of glycemia. The assay was finally validated as a rapid test (15 min) on various clinical samples, showing good reliability and, with further technological development, great potential for future home-testing applications. Overall, it is important to stress that the designed sensing platform could be easily adapted for the monitoring of several other pathologies, directly involving different oxidase enzymes.

## Data Availability Statement

The raw data supporting the conclusions of this article will be made available by the authors upon reasonable request.

## Ethics Statement

The studies involving human participants were reviewed and approved by the Comitato Etico Regionale della Liguria. The patients/participants provided their written informed consent to participate in this study.

## Author Contributions

All the authors contributed to the development of the ideas behind the work, experimental design, and manuscript writing.

## Conflict of Interest

The authors declare that the research was conducted in the absence of any commercial or financial relationships that could be construed as a potential conflict of interest.
