# Supplementary material for: Colorimetric Nanoplasmonics to Spot Hyperglycemia From Saliva
Source: Front Bioeng Biotechnol. 2020 Dec 7;8:601216. doi: 10.3389/fbioe.2020.601216 (PMC7793823; doi:10.3389/fbioe.2020.601216)
Supplement: Supplementary file 1 [file Data_Sheet_1.pdf]

Supplementary Material

Colorimetric nanoplasmonics to spot hyperglycemia from saliva

P. Donati[§], T. Pomili[§], L. Boselli*, and P.P. Pompa*
Nanobiointeractions&Nanodiagnostics, Istituto Italiano di Tecnologia, Via Morego 30, 16163 Genova, Italy

Corresponding authors: luca.boselli@iit.it, pierpaolo.pompa@iit.it

Table of Contents

1 Chemicals and Materials 3
2 Experimental Section 3
2.1 Synthesis of Nanoparticles 3
2.1.1 Synthesis of GNP seeds 3
2.1.2 Synthesis of MGNPs 4
2.2 Nanoparticles Characterization 4
2.2.1 Dynamic Light Scattering analysis 4
2.2.2 Transmission Electron Microscopy analysis 4
2.2.3 UV-visible spectroscopy 4
2.2.4 Reflectance spectroscopy 5
2.3 Experimental procedures for the sensing platform in water 5
2.3.1 Optimization of pH conditions 5
2.3.2 Halogen screening 5
2.3.3 Efficiency of hydrogen peroxide detection 6
2.3.4 Effect of hydrogen peroxide on MGNPs absorption intensity 6
2.3.5 Optimal conditions for the reshaping process in water 6
2.4 Experimental procedures for the sensing platform in saliva 6
2.4.1 Saliva sampling 6
2.4.2 Salivary glucose assay with MGNPs in suspension 7
2.4.3 TEM statistical analysis on MGNP morphological changes (reshaping) in saliva 7
2.5 Glucose dipstick assay prototype 7
2.5.1 Substrate preparation 7
2.5.2 Glucose assay protocol 8
2.5.3 Assay's reliability and validation 8
2.5.4 Assay reproducibility and Limit of detection (LOD) 9
3 Supplementary Figures 10
4 References 22

1 Chemicals and Materials

All chemicals and reagents employed were of high technical grade, stored following vendor recommendations and directly used with no further purification. Hydroxylamine sulfate ($H_3NO•0.5H_2SO_4$, 88944) and Hydrogen Tetrachloroaurate ($HAuCl_4*XH_2O$, Au 49% min, 42803) were purchased from Alfa Aesar. Sodium citrate tribasic hydrate ($HOC(COONa)(CH_2COONa)_2$, ReagentPlus®, ≥99%, 25114), HEPES (BioUltra, for molecular biology, ≥99.5%, 54457), Potassium bromide (KBr,EMSURE® ACS,Reag. 1.04905), Potassium chloride (KCl, EMSURE®,1.04936), Potassium iodide (KI, for analysis EMSURE® ISO,Reag, 1.05043), D-(+)-Glucose (C6H12O6 ACS reagen, G5767) and Glucose Oxidase from Aspergillus (Type X-S, lyophilized powder, G7141) were purchased from Sigma-Aldrich. Nylon membrane filters (Whatman® pore size 0.45 µm, 13 mm Ǿ, thickness150-187 µm, WHA7404001), Nitrocellulose membrane filters (Whatman® pore size 0.45 µm, 13 mm Ǿ, WHA7184001), PDVF Membrane filters (Millipore Durapore®, pore size 0.45 µm, hydrophilic, 13 mm Ǿ, HVLP01300) were purchased from Sigma-Aldrich. Syringe filter holder (re-usable polycarbonate, for 13 mm Ǿ filters, 0.5 cm^2 filtration area, 16514) were purchased from Sartorius. A high sensitivity glucose assay kit (Merck - MAK181-1KT) was employed as reference standard technique. If not specified differently, all solutions and buffers were prepared using ultrapure deionized water (MilliQ). The saliva samples were spontaneously donated from healthy subjects and their use in this study was approved by Ethical Committee. Saliva samples from diabetic patients were provided by the San Matteo hospital of Pavia.

2 Experimental Section

2.1 Synthesis of Nanoparticles

2.1.1 *Synthesis of GNP seeds*

Colloidal 15 nm citrate-capped GNPs were synthesized by Turkevich–Frens method[1],[2] to be used as seeds for MGNPs preparation. Briefly, a trisodium citrate solution (25 mL, 40 mM) was quickly added to a boiling solution of hydrogen tetrachloroaurate (250 mL, 1 mM) under vigorous stirring. After 15 min the red colloidal suspension was cooled down to room temperature, filtered (0.2 µm cellulose acetate syringe filter) and stored at 4 °C. The prepared gold seeds were

characterized by UV-vis spectrophotometry (UV-vis), dynamic light scattering (DLS), and transmission electron microscopy (TEM). The concentration of the colloidal suspension was determined directly from UV-vis spectra, according to *Haiss et al*.[3] previously reported method. Characterization reported in Figure S1.

2.1.2 *Synthesis of MGNPs*

60 nm multibranched gold nanoparticles (MGNPs) were prepared by slightly modified *Maiorano–Pompa* method[4], optimized for our purposes. The procedure relied on a seed-mediated growth. Briefly, 6.5 mL of the prepared 15 nm gold seeds (1.8 nM), 0.28 mL of hydrogen tetrachloroaurate (100 mM) and 0.8 mL of hydroxylamine sulfate (100 mM) were consecutively added to 250 mL of HEPES solution (50 mM, pH = 7.0), under vigorous stirring. After 15-20 minutes at room temperature, the reaction mixture become blue. Residual reagents excess was removed by centrifugation (400 rcf, 25 min). MGNPs were characterized by DLS, UV-vis spectrophotometry and TEM. Characterization reported in Figure S2. The optimization of shape and plasmonic feature was performed by varying the reagents stoichiometry.

2.2 Nanoparticles Characterization

2.2.1 *Dynamic Light Scattering analysis*

Nanoparticle hydrodynamic diameter (Dh) was measured by Zetasizer Nano Range (Malvern Panalytical) as frequency distribution of intensity, size and volume. Reported values are averaged results from 3 measurements (each consisting of an accumulation of 11 runs).

2.2.2 *Transmission Electron Microscopy analysis*

The TEM analysis was performed using a JEOL JEM 1400 microscope. Samples were prepared drop casting 3 µL of each solution on oxygen plasma cleaned grid (CF150-Cu-50 - Carbon Film 150 Mesh, Cu, 50/bx) and vacuum drying.

2.2.3 *UV-visible spectroscopy*

UV-vis spectra (400-800 nm) of the nanoparticle suspension were acquired by a Thermo Fisher NanoDrop® (wavelength Accuracy ± 1 nm, absorbance accuracy 3 % at 0.74 Abs@350nm) with

a small volume cuvette or by Tecan Spark® multimode microplate reader (wavelength accuracy < 0.3 nm, absorbance accuracy < 0.5%@260) nm with flat transparent 96 multiwell plate.

2.2.4 Reflectance spectroscopy

Reflectance spectra were recorded on dried substrates using Ocean Optics spectrophotometer equipped with a reflection probe and an OCEAN-HDX-XR detector. For the white balance, a reflectance standard was employed (Diffuse Reflectance Std, Spectralon).

2.3 Experimental procedures for the sensing platform in water

2.3.1 Optimization of pH conditions

Different acetate buffers (14 mM) were prepared varying the ratio between acetic acid and sodium acetate to obtain three solutions with pH 4.5, 5.0 and 5.5, respectively.
20 µL of MGNPs (0.5 nM, A_{LSPR} = 0.8, ε = 16.87 × 10^9 $M^{-1}cm^{-1}$), 20 µL of KBr (50 mM) and 20 µL of H_2O_2 (2.5 mM) were added to 140 µL of the different acetate buffer solutions (pH = 4.5, 5.0 and 5.5). Ctrls were performed in absence of H_2O_2 (replaced by 20 µL of H_2O).
The experiment was performed in a 96-multiwell plate and absorption spectra were recorded at room temperature by Tecan plate reader to monitor the reaction over time (see Figure S3).

2.3.2 Halogen screening

Three halogen ion solutions were used at their "best" operational concentration (previously experimentally identified), since, due to their very different reactivity, employing them at the very same concentration would have not allowed for a fair comparison among their potential role in the sensing platform.
KCl (500 mM), KBr (50 mM) and KI (50 µM) were the selected stock solutions used in this study. 20 µL of MGNPs (0.5 nM, A_{LSPR} = 8.0, ε = 16.87 × 10^9 $M^{-1}cm^{-1}$), 20 µL of acetate buffer (100 mM, pH = 5) and 20 µL of halogen stock solution were added to 140 µL of H_2O_2 (2.86 mM). H_2O_2 final concentration was 2 mM in the total volume. See Figure 1b (main text).

2.3.3 *Efficiency of hydrogen peroxide detection*

Several "sample" solutions with different H_2O_2 concentrations were prepared (0, 0.71, 2.14, 3.57 and 4.29 mM).

20 µL of MGNPs (0.5 nM, A_{LSPR} = 0.8, ε = 16,87 ×10^9 $M^{-1}cm^{-1}$), 20 µL of KBr (50 mM) and 20 µL of acetate buffer (100 mM, pH = 5) were added to 140 µL of the prepared H_2O_2 solutions (presenting, in the total volume, a final concentration of 0, 0.5, 1.5, 2.5 and 3 mM).

The experiment was performed in a 96-multiwell plate and absorption spectra were recorded at room temperature by Tecan plate reader to monitor the reaction over time (see Figure S4).

2.3.4 *Effect of hydrogen peroxide on MGNPs absorption intensity*

The experimental procedure was carried out as described on the previous section but involving H_2O_2 solutions final concentration 1, 3, 5, 10, 20 and 30 mM (see Figure S5).

2.3.5 *Optimal conditions for the reshaping process in water*

20 µL of MGNPs (0.5 nM, A_{LSPR} = 0.8, ε = 16,87 ×10^9 $M^{-1}cm^{-1}$), 20 µL of KBr (50 mM) and 20 µL of acetate buffer (100 mM, pH = 5) were added to 140 µL of H_2O_2 solution presenting, in the total volume, a final concentration of 3 mM. See Figure 1d (main text).

2.4 Experimental procedures for the sensing platform in saliva

2.4.1 *Saliva sampling*

The saliva samples were spontaneously donated from healthy subjects, and their use in this study was approved by Ethical Committee.

Donors were asked to avoid eating, drinking and oral hygiene procedures for at least one hour before saliva collection. Furthermore, donors were asked to provide unstimulated saliva (no speaking or swallowing for one minute prior to collection). Samples were collected by the donor into a 15 mL sterile and protein lo-bind falcon tube. The tube was stored at 4 °C and processed within one hour from donation.

The sensing platform was conceived for self-monitoring of untreated whole saliva, however in this work we employed saliva samples from different donors and therefore, for safety reasons, samples were manipulated under the chemical hood and filtered (0.2 µm methyl cellulose syringe

filter) from bacteria. Saliva samples from diabetic patients were provided by the San Matteo hospital of Pavia.

2.4.2 Salivary glucose assay with MGNPs in suspension

Hyperglycemia conditions were simulated by supplementing the collected saliva samples (see the saliva sampling section) with a glucose solution in order to reach pathological concentrations (2 mM, 36 mg/dL). Non-supplemented saliva representing physiological normal condition was used as a control.

20 µL of MGNPs (0.5 nM), 20 µL of GOx (0.021 mg/mL, 3 U/mL) in acetate buffer (50 mM, pH = 5), and 80 µL of KBr solution (5 M) were added to 80 µL of glucose supplemented saliva under stirring at 37 °C. See Figure S7 and Figure 2b (main text).

2.4.3 TEM statistical analysis on MGNP morphological changes (reshaping) in saliva

The glucose assay was performed as described in the previous section. The mixture was stirred at 37 °C for 10 min and, after colour change from blue to red, the reaction was stopped diluting and washing (centrifugation and resuspension process) the nanoparticles with cold (4 °C) HEPES buffer (10 mM, pH = 8.0). The ctrl experiment was performed using non-supplemented saliva.

The obtained nanoparticles were analysed by TEM. The dimensional analysis results are presented in Figure 2 (main text). Histogram shows the comparison of measured dimensions as number (N = 320) and length of tips (N = 1700) and core diameter (N = 1400). All samples are normally distributed (as also dot plot show) while Mann-Whitney test confirm statistical significance (p-value < 0.001) of mean sizes distribution difference (see Figure 2 main text).

2.5 Glucose dipstick assay prototype

2.5.1 Substrate preparation

0.4 mL of a freshly prepared MGNP suspension (25 pM, A_{LSPR} = 0.4) in HEPES buffer (25 mM, pH = 7.0) were passed through a 0.4 µm porous nylon membrane using a syringe filter holder (see Figure S). The system was maintained in a vertically position promoting the uniform injection of the liquid and therefore uniform immobilization of the MGNPs on the membrane. Subsequently, 2 mL of air were injected to remove any residual water from the system. The prepared substrate was dried under vacuum for 10 minutes and then 15 µL of GOx solution (1 mg/mL, 145 U/mL) in

acetate buffer (100 mM, pH = 5) were deposited by direct drop casting on the membrane. The coated nylon support was dried again under vacuum and, finally, stored in low humidity conditions.

2.5.2 *Glucose assay protocol*

The glucose tests were performed adding on the prepared substrate 50 µL glucose supplemented saliva (90 – 180 µM, 1.5 – 3 mg/dL) and 50 µL of KBr solution (5 M) in acetate buffer (10 mM, pH = 5). The tests were carried out incubating the supports at 37 °C for ca. 15 minutes. The test can be also carried out at room temperature leading to the same outcome but requiring a slightly longer time window.

2.5.3 *Assay's reliability and validation*

The glucose concentration of twenty saliva samples (eight from healthy subjects and twelve from diabetic subjects) was measured using a high sensitivity glucose assay kit (Merck - MAK181-1KT) chosen as a standard reference technique.

Six saliva samples from healthy subjects presenting a physiological glucose concentration < 2 mg/dL were selected to numerically estimate the colorimetric changes of our device at a targeted threshold (4 mg/dL ± 0.5).

The glucose-dependent assay's color change was estimated after 15 min using RGB coordinates acquired using ColorGrab (smartphone app). The ΔRGB (see formula below) obtained at different glucose concentrations was employed for statistical data analysis:

$$\Delta RGB = \sqrt{(R_{t1} - R_{t0})^2 + (G_{t1} - G_{t0})^2 \ + (B_{t1} - B_{t0})^2}$$

Glucose spikes were employed to normalize the saliva samples to 2.5, 4, and 6 mg/dL to evaluate the ΔRGB variability and the ΔRGB value range, allowing to identify our threshold.

An evident color change is expected for salivary glucose concentrations ≥ 4 ± 0.5 mg/dL after 15 min of test was estimated to correspond approximately to ΔRGB ≥ 30 ± 10 while little or no color change is expected for physiological concentrations (ΔRGB < 15 ± 5).

Altogether, the twenty saliva samples were analyzed for their basal content using our ON/OFF colorimetric assay and the commercial kit to perform a small clinical trial (see Figure 4).

2.5.4 Assay reproducibility and Limit of detection (LOD)

The experiments were performed using saliva samples from healthy donors, and the basal glucose concentration was measured using a high sensitivity glucose assay kit (Merck - MAK181-1KT) as a reference technique.

The device reproducibility was tested using five independently produced assays, prepared with the same protocol. The reagents ratios were tuned to obtain an evident color change for salivary glucose concentrations ≥ 4 ± 0.5 mg/dL as the selected threshold. The reproducibility tests were performed using different glucose concentrations and identifying the color change numerically by extracting the RGB coordinates using ColorGrab (smartphone app). The acquired ΔRGB obtained at different glucose concentrations in after 15 minutes of test were also employed to calculate the LOD See graphs in Figure S12.

The LOD was calculated based on the ratio between three times the standard deviation for the control and the angular coefficient obtained from the analytical curve (see formula below).

$$X_{LOD} = 3.3*SD_{pseudo\text{-}blank}/b$$

X_{LOD}: limit of detection; $SD_{pseudo\text{-}blank}$: standard deviation of the pseudo-blank (non-supplemented saliva); b: slope of the calibration .

Further LOD calculations involving six different saliva samples from healthy subjects presenting a basal glucose concentration < 2 mg/mL were performed. The calculated average LOD was 1.4 mg/dL.

3 Supplementary Figures

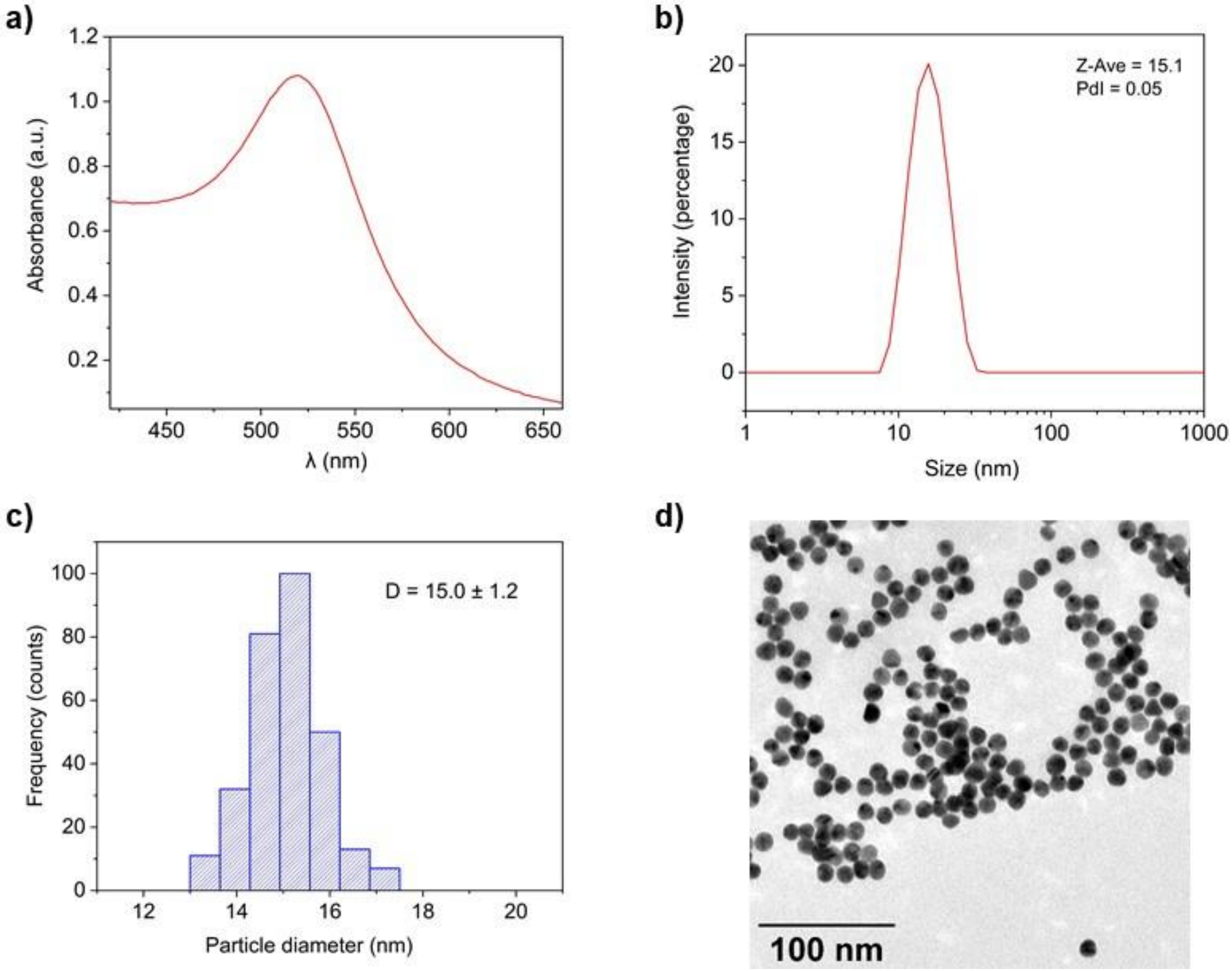

Figure S1. GNP seeds characterization*: a) UV-vis absorption spectrum in water; b) DLS measurement (Dh = 15 nm); c) TEM size distribution analysis showing a monodisperse population with an average size of 15 nm; d) representative TEM micrograph (scale bar: 100 nm).*

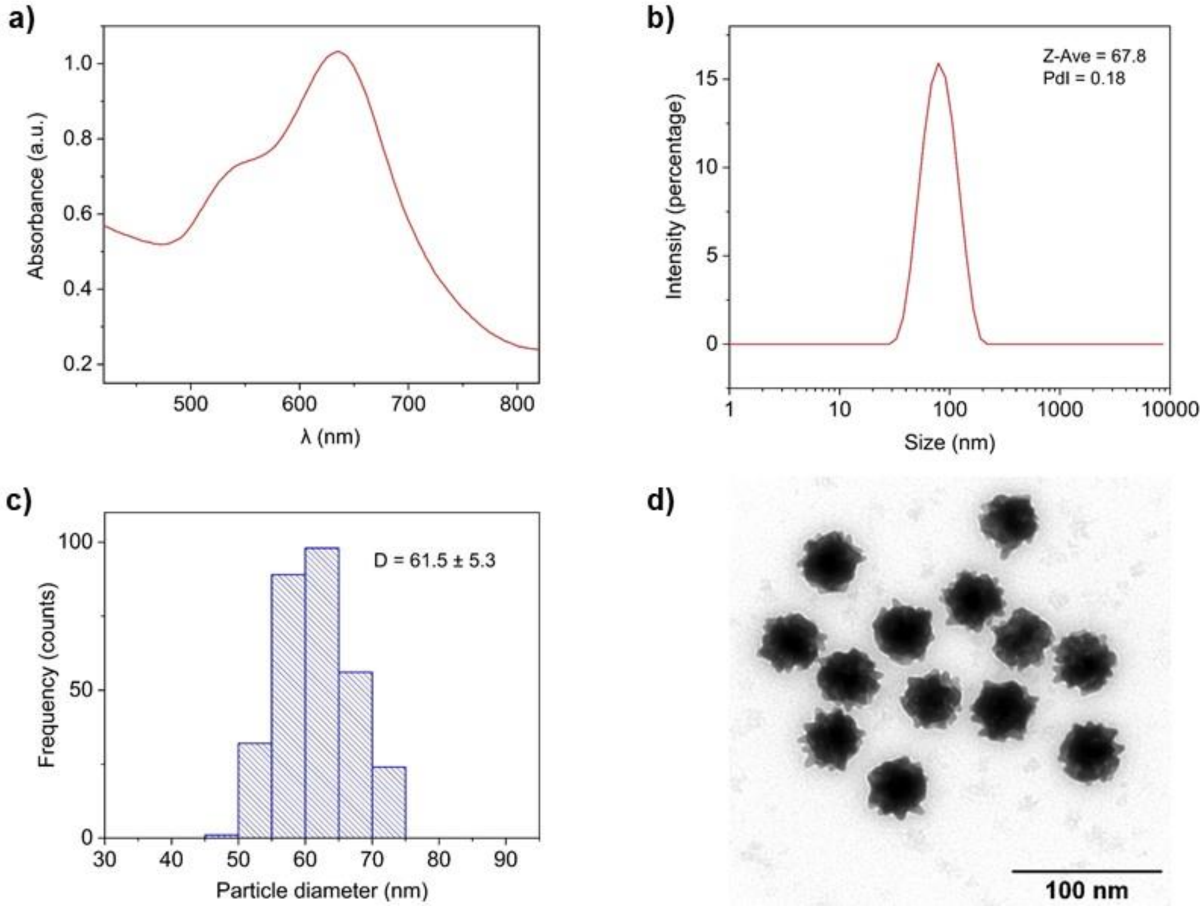

Figure S2. MGNP characterization*: a) UV-vis absorption spectrum in water; b) DLS measurement (Dh = 68 nm) c) TEM size distribution analysis showing a monodisperse population with an average size (longest tip to tip distance) of about 61 nm; d) representative TEM micrograph (scale bar: 100 nm).*

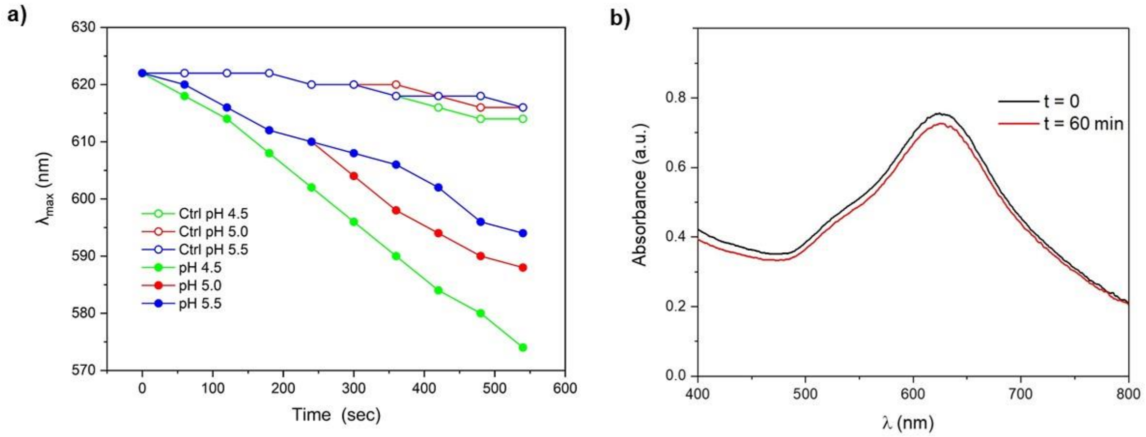

***Figure S3. Optimization of pH conditions**: a) LSPR λ_{max} evolution over time related to MGNPs reaction with H_2O_2 and KBr at pH = 4.5 (green line), pH = 5 (red line) and pH = 5.5 (blue line). Remarkable LSPR λ_{max} shift in less than 10 min can be observed for all tested conditions. Decreasing the pH led to faster and wider blue-shift. Ctrls were performed in absence of H_2O_2 showing little LSPR λ_{max} variations. For more details see experimental procedure section. b) Stability test of MGNPs in acetate buffer pH = 5: the spectra show good colloidal stability over 60 min;*

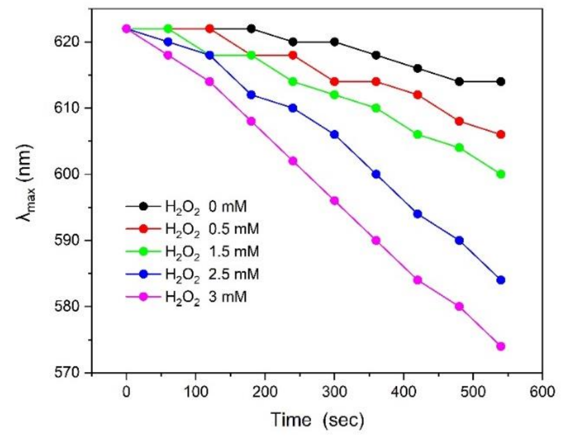

***Figure S4. Efficiency of hydrogen peroxide detection in water**: LSPR λ_{max} evolution over time related to MGNP reaction with increasing H_2O_2 concentration (in presence of KBr at pH = 5). For more details, see experimental procedure section. H_2O_2 spectroscopically detectable within 10 min at all tested concentrations. Increasing the H_2O_2 concentration the LSPR λ_{max} shift became faster and wider.*

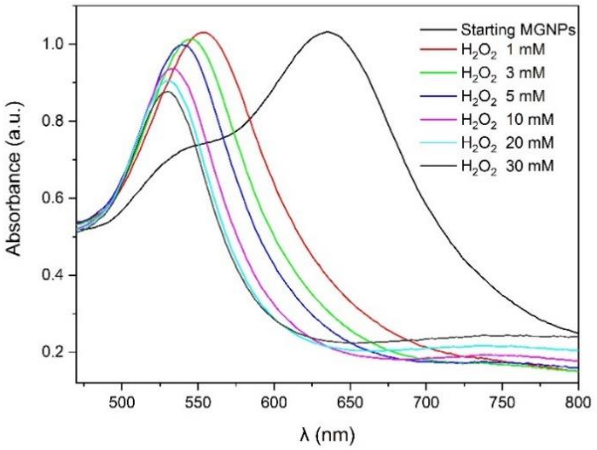

***Figure S5. Effect of hydrogen peroxide on MGNP absorption intensity in water**: absorption spectra related to MGNPs before (starting MGNP) and after reaction with H_2O_2 at increasing concentrations (in presence of KBr at pH = 5). To ensure a comparison between completed reactions, the spectra were measured after 90 min. While H_2O_2 at 1-3 mM induced minimal absorption intensity variation, at higher concentrations (10-20-30 mM) significant loss of LSPR intensity was observed, suggesting that etching process occurred.*

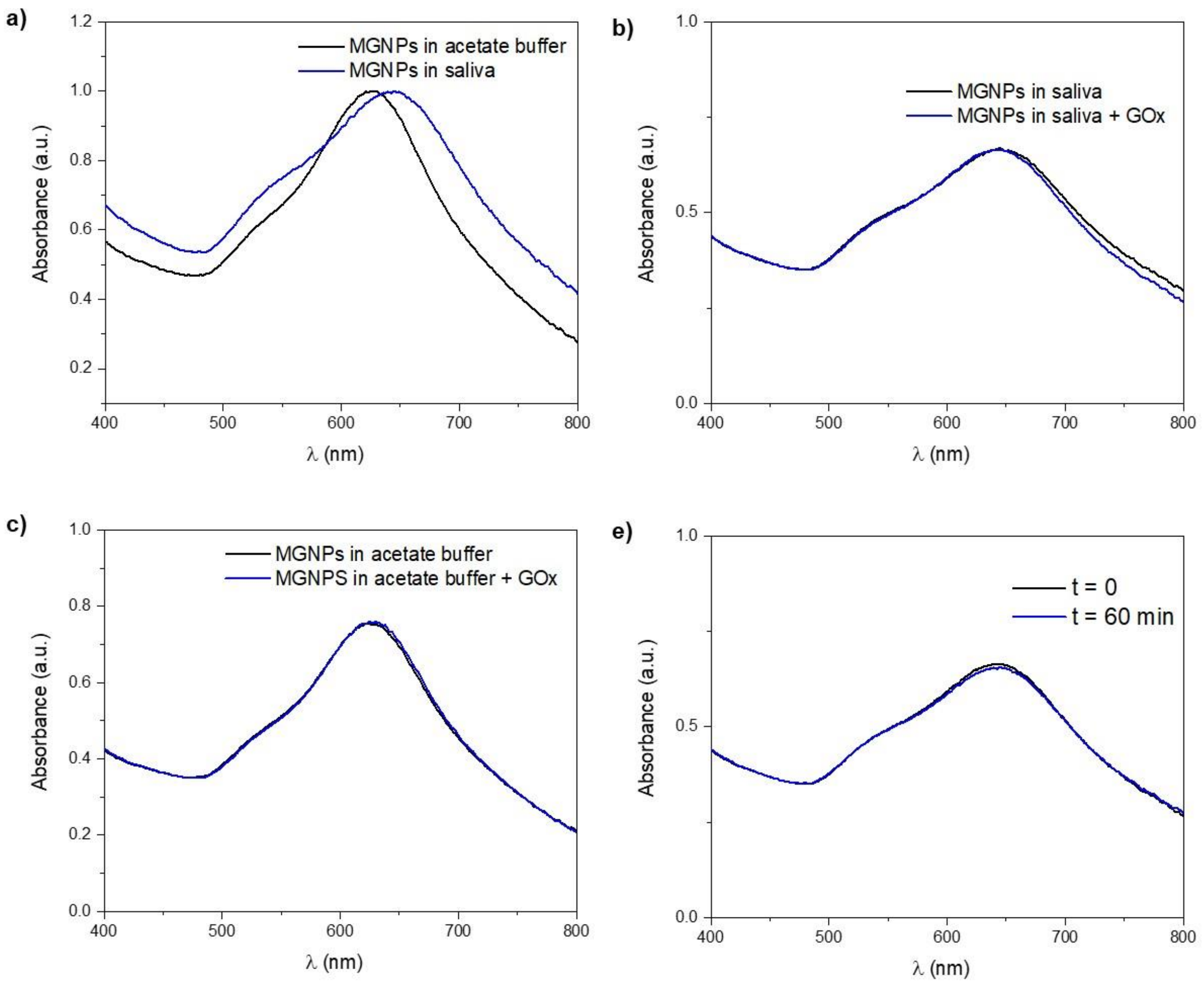

Figure S6. Protein corona and MGNP stability in saliva. *a) Characteristic LSPR red-shift related to the protein adsorption on plasmonic nanoparticles. b) and c) show no GOx effect on the LSPR. e) Stability test of MGNPs in saliva: the absorption spectra show good colloidal stability over 60 min also in presence of the enzyme. The saliva employed was previously diluted 1:4 in acetate buffer as for assay condition.*

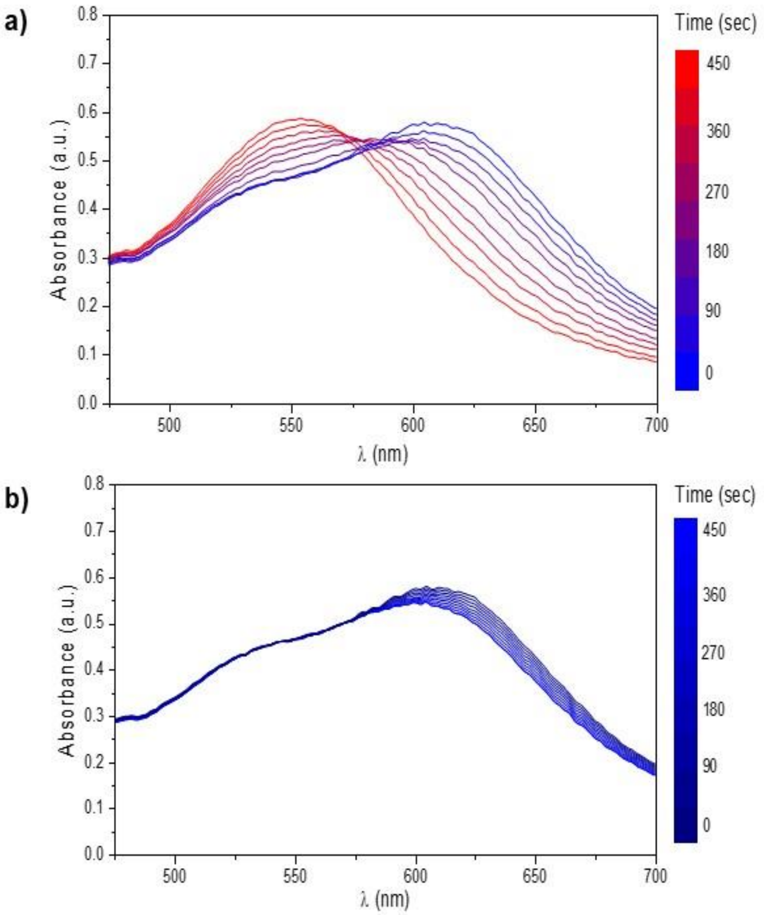

Figure S7. Reshaping process in saliva: *MGNP absorption spectra evolution over time during the assay using a) glucose supplemented saliva (hyperglycemic condition) b) physiological saliva (ctrl). For more details, see the experimental procedure section.*

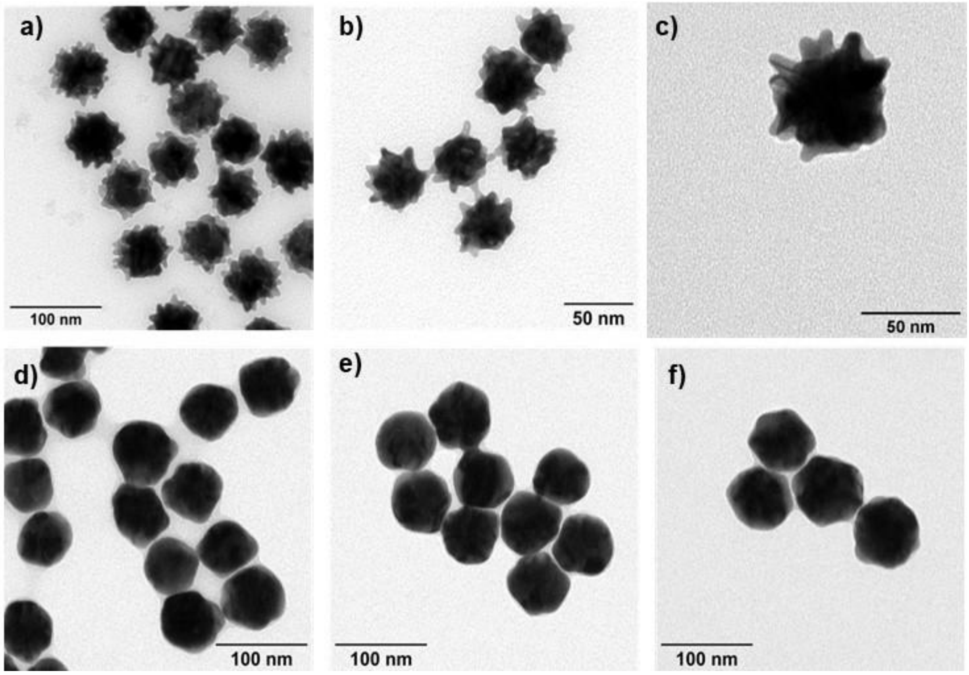

Figure S8. TEM images of MGNPs after glucose assay in saliva*: a)-c) representative images of the MGNPs after testing non-supplemented saliva sample (physiological Ctrl); d)-f) representative images of MGNPs after testing glucose-supplemented saliva (hyperglycemia condition). While the ctrl did not exhibit significant morphological changes, the saliva sample with pathological concentration of glucose led to nanostructures rearrangement from multibranched to spherical shape.*

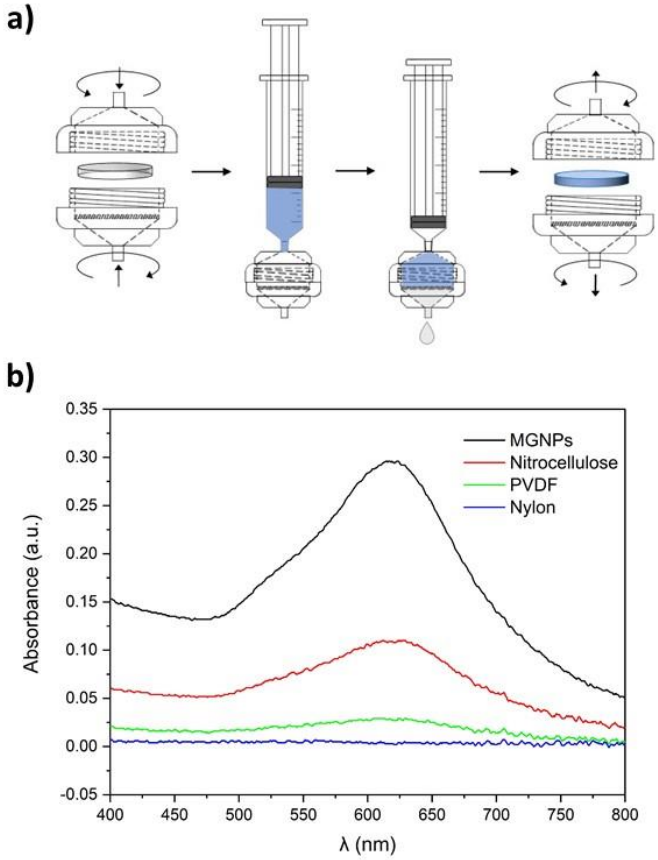

Figure S9. Preparation of the membrane substrate and MGNP retention screening*: a) schematics of the MGNP deposition on the membrane substrate. b) UV-vis absorption spectra of a MGNPs (20 pM) aqueous solution before (black curve) and after filtration through different membranes (nitrocellulose, PVDF and nylon). All the MGNPs were retained in the nylon membrane, leading to colorless filtrate (no light absorption, blue curve).*

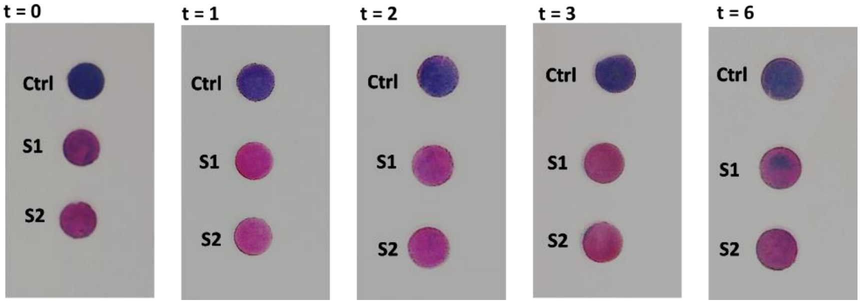

Figure S10. Stability studies of the glucose assay prototype over time: *the pictures show the color change of the substrate after 15 min of assay. Different samples were tested including saliva with no additional glucose (Ctrl) and supplemented with glucose (S1 = 1.5 mg/dL and S2 = 3 mg/dL) to verify that the detection limit of the prototype was not compromised over time. Stability of the system was confirmed over 6 months.*

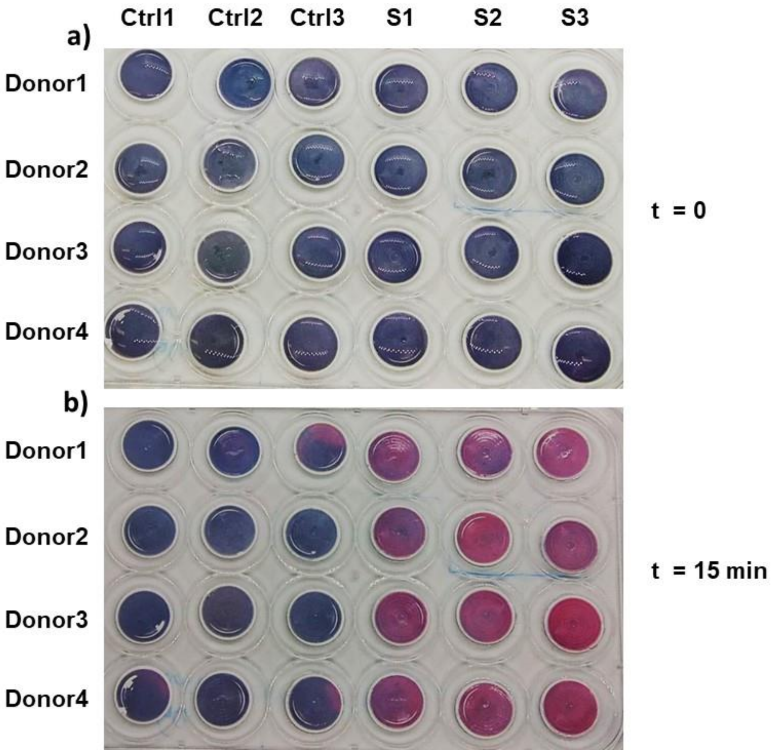

Figure S11. Preliminary validation of the glucose assay prototype on different saliva samples*: picture of the glucose assay a) immediately after saliva sample addition on the membrane and b) after 15 min. The saliva samples collected from different donors were tested with no additional glucose (Ctrl3) and supplemented with glucose (S1 = 1.5 mg/dL, S2 = 3 mg/dL, S3 = 5 mg/dL) to reproduce hyperglycemic conditions. Additional controls in which the assay was performed on non-supplemented saliva but in absence of GOx (Ctrl1) or in presence of denaturated GOx (Ctrl2) were also included in the study.*

a)

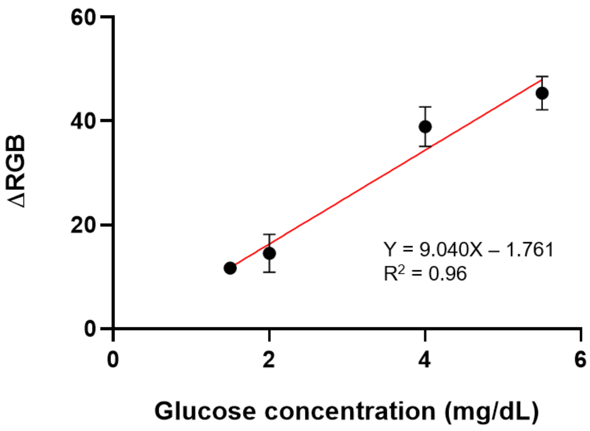

b)

Donors	ΔRGB
1	8
2	32
3	58
4	34
5	16
6	13
7	56
8	64
9	6
10	22
11	11
12	18
13	10
14	7
15	8
16	12
17	12
18	24
19	8
20	13

Figure S12. Assay performance analysis: *a) Analytical plot for LOD calculation; b) ΔRGB data relative to the color change of the assay obtained from clinical samples.*

4 References

[1] J. Turkevich, P. C. Stevenson, J. Hillier, *Discuss. Faraday Soc.* **1951**, *11*, 55–75.

[2] J. E. B. A.J.Clarke, *Nat. Phys. Sci.* **1973**, *241*, 20.

[3] W. Haiss, N. T. K. Thanh, J. Aveyard, D. G. Fernig, *Anal. Chem.* **2007**, *79*, 4215–4221.

[4] G. Maiorano, L. Rizzello, M. A. Malvindi, S. S. Shankar, L. Martiradonna, A. Falqui, R. Cingolani, P. P. Pompa, *Nanoscale* **2011**, *3*, 2227–2232.
